# A Smile Reimagined: Gummy Smile Resolution with Cutting Edge Method Using Chu's Gauge and Polymethylmethacrylate (PMMA) Bone Cement

**DOI:** 10.1155/2024/6500762

**Published:** 2024-06-06

**Authors:** Shalini Mundra, Neetha. J. Shetty

**Affiliations:** ^1^ Department of Periodontology Manipal College of Dental Sciences, Mangalore, India; ^2^ Manipal Academy of Higher Education (MAHE), Manipal, India

## Abstract

Excessive gingival display (EGD) is one of the most common aesthetic concerns, and its correction often presents a challenge to periodontists. It has a multifactorial etiology, and this article describes a case involving hypermobile upper lip (HUL), altered passive eruption (APE), and vertical maxillary excess (VME). Upon investigation, a positive collum angle and marked subnasal skeletal depression were observed. In this context, it is noted that during a spontaneous smile, the upper lip retracts and gets lodged in this depression. The rehabilitation plan includes included aesthetic crown lengthening via gingivectomy using Chu's proportional gauge for altered passive eruption and filling the subnasal depression by PMMA (polymethylmethacrylate) bone cement. The entire treatment plan was digitalised using cutting edge methods such as computed tomography (CT), cone beam computed tomography (CBCT), and 3D printers for virtual planning of the defect's position, size, and shape. No postoperative complications were reported. After six months, the patient exhibited a harmonious smile with reduced exposed gingiva.

## 1. Introduction

Smile is one of the most powerful communication tools. Enhancing one's smile and appearance has become the mantra of the maturing “baby boomer” population. A common request from today's sophisticated patients is correction of excessive gingival display (EGD) or gummy smile. The challenge for modern dentists is to balance aesthetic and functional objectives while respecting biological parameters. As aesthetic-minded periodontal surgeons armed with periodontal plastic procedures, we can happily fulfil the patient desires [1].

The crafting of an ideal smile necessitates an analysis of both the hard and soft tissues comprising the face and smile. In dentistry, there are three basic tenets germaine to optimal aesthetics categorised as follows: (a) facial aesthetics (encompassing facial height, shape, profile, gender, and age), (b) dental composition (referring to size, shape, and position of the teeth), and (c) dentofacial composition (including lip morphology, mobility, position, and the smile's relation to the face) [2]. Among all these components, lip analysis stands out as the most critical. Any asymmetry in this parallelism disturbs the harmony, resulting in an unaesthetic gummy smile.

Excessive gingival display, also known as gummy smile, is a multifactorial condition that can result from various factors such as altered passive eruption (APE) [3, 4], hypermobile upper lip (HUL) [5], craniofacial deformities like vertical maxillary excess, and short clinical crown [6]. Among all the established etiologies, altered passive eruption and hypermobile upper lip are the most prevalent. APE and HUL can coexist in the same patient, leading to greater gingival display than either etiology alone [7, 8]. Therefore, it is crucial to identify this etiology before initiating any rehabilitation treatment [9, 10].

A multidisciplinary approach consisting of clinical crown lengthening [11], lip repositioning [12], botulin toxin injections [13], and use of fillers like PMMA ([14, 15] is often considered for correcting EGD. However, in the present article, the etiology for EGD involves a combination of skeletal dysplasia (vertical maxillary excess), hypermobile upper lip, and altered passive eruption. Therefore, for such challenging cases with multifactorial etiology, a more holistic approach is imperative. The amalgamation of aesthetic clinical crown lengthening with Chu's proportional gauge and lip repositioning using a filler material like polymethylmethacrylate (PMMA) bone cement can lead to more aesthetic and predictable results.

## 2. Case Report

**Figure 1:**
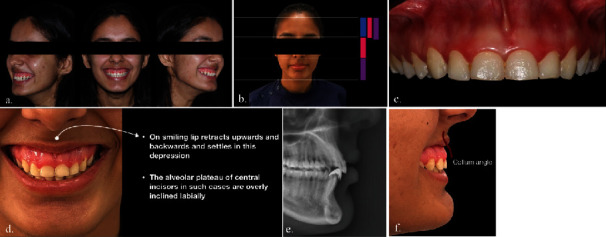
(a) Preoperative front and lateral profile. (b) Extraoral examination: face analysis. (c) Intraoral examination: shorter clinical crown. (d) Lip analysis. (e–f) Radiographic examination: collum angle (the supplementary angle of the crown-root angle).

A 23-year-old female presented to the Department of Periodontology with the chief complaint of excessive gingival display while smiling. The patient had undergone orthodontic treatment 10 years prior and expressed dissatisfaction with the outcome. There was no relevant medical or family history.  Figure 1(a) shows the preoperative front and lateral profiles of the patient. The extraoral examination (Figure 1(b)) revealed vertical maxillary excess with larger measurements in the lower 1/3 of the face. Lip analysis (Figure 1(d)) revealed a hyperactive upper lip with 10 mm of muscular activity, along with an 8 mm gingival display from one side of the maxillary molar to the opposite side during smiling. Intraoral examination (Figure 1(c)) did not reveal any soft tissue abnormalities. The clinical crown width-to-length ratio relative to teeth 16-26 exceeded the ideal width-to-length ratio, indicating short clinical crowns (more than 75-80%). Radiographic examination (Figures 1(e) and 1(f)) showed a positive collum angle between alveolar bone plateau and central incisors.

**Figure 2:**
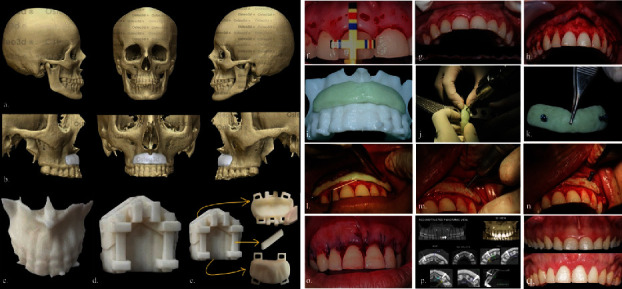
Digital planning: (a) computed tomography (CT) scan; (b) virtual planning to plan position, size, and shape of PMMA block; (c) prototype of cropped maxillary segment for test fit; (d) prototype of mould for fabrication of PMMA block; (e) parts of 3D printed mould. Surgical intervention: (f) bleeding points are marked using Chu's aesthetic gauge; (g) gingivectomy is performed; (h) full thickness flap elevated; (i) passive adaptation of PMMA block checked by placing over printed maxillary segment; (j) screw positions are marked, and two holes are drilled of 2 mm diameter using bone drills; (k) extraoral placement of titanium screw over the prepared PMMA block; (l–m) PMMA block placement; (n) fixation of PMMA block using titanium screws; (o) flap adaptation using sling sutures; (p) postoperative CBCT to confirm the position of PMMA block; (q) pre- and postoperative intraoral photographs.

Additionally, a computed tomography (CT) scan (Figure 2(a)) was performed to evaluate alveolar bone anatomy and for virtual planning of a PMMA block. According to 2017 AAP-EFP classification, the diagnosis was excessive gingival display associated with a hyperactive upper lip, altered passive eruption type I-A, and vertical maxillary excess. Informed consent was obtained from the patient, and the article followed the CARE guidelines.

## 3. Case Management

### 3.1. Digital Planning and 3D Printing of Models

A CT scan was performed to plan the position, size, and shape of the PMMA block. The digital planning process involved submitting the scan data to “Osteo3d – 3D Printing Healthcare” in Bangalore, India, to generate prototypes of the defect. Virtual planning assisted in achieving an optimal fit of the block to the defect geometry and aided in planning the position of screws to prevent any interference with vital structures or tooth roots. After confirmation and approval for production, the final design was printed using a 3D printer. The prototypes developed included (i) a cropped maxillary segment for test fitting and (ii) a mould for the fabrication of the PMMA block (Figures 2(b), 2(c), 2(d), and 2(e)).

### 3.2. Manipulation of PMMA PALACOS R+G Bone Cement

**Figure 3:**
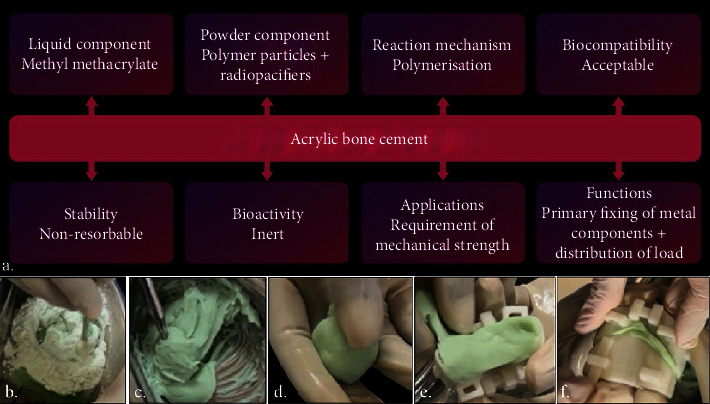
(a) PMMA bone cement properties. (b–f) Bone cement manipulation and adaptation in the 3D printed mould.

The manipulation of PMMA bone cement followed the manufacturer's instructions. The cement was mixed in a sterile surgical bowl. When the mixture reached the dough stage, indicated by no longer sticking to the gloves and becoming easily adaptable, it was transferred into the 3D printed mould (Figures 3(b), 3(c), 3(d), 3(e), and 3(f)). Constant pressure was applied throughout the polymerization process.

### 3.3. Surgical Procedure

#### 3.3.1. Crown Lengthening with Chu's Aesthetic Gauge

The surgery was performed under local anesthesia. Bleeding points were established at the zenith point with the aid of Chu's proportional gauge [16]. The T-bar tip of the proportional gauge provides quick and accurate diagnosis of tooth proportion as it measures both length and width of the tooth. Gingivectomy was carried out using a #15C blade. Subsequently, intrasulcular incisions were made, and a full thickness flap was raised to expose the bone. The T-bar tip of the proportion gauge was then replaced with the Biologic Periogauge (BLPG) tip [16]. This assists in achieving the desired biologic crown length and allows for osteoplasty where necessary using spherical burs at high speed under copious saline irrigation (Figures 2(f), 2(g), and 2(h)).

The prepared PMMA block was verified for passive adaptation by placing it on cropped maxillary segment. Later, at the predetermined position (i.e., one drill between teeth 12 and 13 and the other between teeth 22 and 23), two holes were placed in the PMMA block using bone drills (Figures 2(i), 2(j), 2(k), 2(l), and 2(m)). The prepared block was stabilised using two titanium-based bone graft fixation screws, each measuring 2∗8 mm, using a screwdriver at the subnasal depression. The flap was repositioned with sling sutures (Figures 2(n) and 2(o)). Immediately after surgery, a CBCT was taken to check the final screw position (Figure 2(p)).

Postoperatively, patient was prescribed analgesics (diclofenac and serratiopeptidase (Tab Lyser-D)) twice daily for 2-3 days, along with an antibiotic (1000 mg amoxicillin and clavulanic acid (Cap Augmentin)) for 7 days to prevent infection. The patient was advised not to brush the surgical site for 14 days and were instructed to use a 0.2% chlorhexidine digluconate mouth rinse twice daily for 14 days postoperatively. Suture removal was scheduled for14 days after surgery. At that time, the patient was instructed on mechanical tooth cleaning using an ultrasoft manual toothbrush with roll technique, gradually returning to regular oral hygiene measures at 1-month postsurgery.

## 4. Clinical Outcome

**Figure 4:**
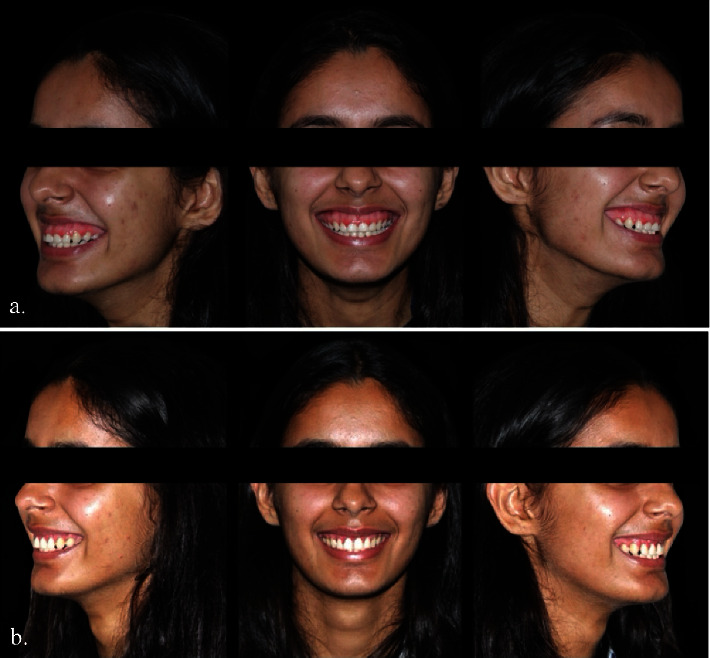
(a) Pre- and (b) postoperative front and lateral profile of the patient.

At the 6-month follow-up, it was observed that the patient had achieved a harmonious smile, a reduction in exposed gingiva, and repositioning of the upper lip (Figure 2(q)).  Figure 4 displays the pre- and postoperative front and lateral profiles of the patient.

## 5. Discussion

In the present article, excessive gingival display results from a combination of hypermobile upper lip, altered passive eruption, and vertical maxillary excess. The primary treatment approach for such combined cases should aim to address discrepancies between soft and hard tissues, achieving a more aesthetically pleasing tooth silhouette [2]. This treatment successfully eliminates any component of altered passive eruption, leaving only vertical maxillary excess visible. Subsequently, the severity of vertical maxillary excess is assessed based on the remaining amount of gingiva, guiding treatment planning accordingly [6]. It is important to note in this context that the alveolar plateau of the central incisors is excessively inclined labially, resulting in a positive collum angle [17, 18].

In our case report, we initially performed external bevel gingivectomy to address altered passive eruption. The primary treatment for vertical maxillary excess degree III typically involves a combination of orthognathic surgery periodontics and restorative dentistry as needed. However, orthognathic surgery presents several challenges including surgical complications, lengthy recovery periods, the need for postoperative orthodontic adjustments, costs, risk of relapse, and potential emotional and psychological impacts on the patient. To mitigate these drawbacks, we crafted an alternative approach.

The alternative option involves the placement of a PMMA bone cement implant in the subnasal depression. This restricts upper lip movement in the upward and backward directions, aiding in reducing gingival exposure. It is worth noting that the use of fillers like PMMA implants is not technically considered as lip repositioning procedure but rather an additional means to minimise muscle movement during smiling [10]. The application of PMMA-based bone cement to fill the subnasal depression was first described by de Torres et al. [19], where the authors manipulated the cement intraoperatively and adapted it directly onto the bone surface with no complications.

Polymethylmethacrylate (PMMA) bone cement [20], commercially available as PALACOS R+G bone cement (Heraeus Medical GmbH, Wehrheim, Germany), is a cross-linked polymer consisting of powder and liquid components. The properties of PMMA (Figure 3(a)), such as its biocompatibility, rigidity, inertness, and cost effectiveness, make it suitable for biomedical applications. The constituents of PMMA bone cement are listed in Table 1. PMMA is widely used in cranioplasty, hip/knee replacement surgeries, and in the fixation of mandibular fractures.

The material is relatively safe; however, the curing process of PMMA involves an exothermic reaction. Consequently, during the setting of PMMA, varying degrees of heat are released. Intraoperative complications arising from high temperatures include tissue damage, potential triggering of the inflammatory cascade, and necrosis of adjacent tissues [19]. To mitigate these complications, 3D printed moulds and models are utilized, which are prefabricated based on tomographic data. These moods serve as templates onto which PMMA bone cement is adapted to achieve a uniform PMMA block. The resultant block can be assessed extraorally by placing it over the printed maxillary model.

To further optimise the handling of exothermic reaction during PMMA fixation, constant irrigation with saline is recommended. This ensures a wet interface between the cement block and tissues. Preventing tissue necrosis in case the curing is incomplete. Additionally, it aids in the removal of residual monomer, preventing potential toxicity such as aseptic loosening of the PMMA block due to bone resorption around the PMMA cement caused by the residual monomer.

Another crucial point is that the amount of heat released correlates positively with the thickness and volume of the prepared PMMA block. It has been observed that as the PMMA block thickness increases, more heat is released. In cranioplasty, a thickness of 7 mm is recommended as safe to work with when manipulated and shaped intraoperatively. However, in the present case where the objective is to fill a gap, a thickness of 3-4 mm is optimal.

Among the complications, Kim et al. [21] reported a long-term infection rate of 13.9%, while Cheng et al. [22] reported infections in 6.25% of cases in cranioplasty. In contrast, Rotaru et al. [23] utilized 3D custom-made cranial implants obtained from tomographic data and rapid prototyping, reporting no complications. Freitas De Andrade et al. [15] prefabricated PMMA bone cement using a 3D printer (Objet V260) for excessive gingival display. The advantages of prefabricating the filler include no risk of tissue damage as polymerization is completed, time savings during surgery, and easy accessibility.

Regardless of the approach used, a lack of proper treatment planning or technical execution can lead to poorer outcomes and/or aesthetic compromises. The aesthetic benefits of present technique are multifaceted. Firstly, the digital workflow includes CBCT and CT scans, intra- and extraoral digital photographs, and 3D printing models. Secondly, the patient-specific tailored treatment plan is implemented, which includes prefabrication of PMMA bone cement block on 3D printed maxilla model. Thirdly, precise incisions for gingivectomy were planned using Chu's aesthetic gauge, leaving no room for error or postcomplications of aesthetic crown lengthening. Fourthly, the use of fillers like PMMA reduces complications associated with lip repositioning surgery, such as tension during smiling or speaking, slight scarring at the coronal incision line, and in few cases relapse.

In conclusion, the key to successfully managing such an aesthetically demanding case lies in proper selection and diagnosis, precise digital planning, along with skilled surgical execution, and adherence to the workflow. However, further studies are necessary to achieve more significant results regarding the outcome and long-term stability of this procedure.

## Figures and Tables

**Table 1:** Constituents of PMMA bone cement.

Powder			Liquid		
1	Polymer	Polymethyl methacrylate/copolymer (PMMA)	1	Monomer	Methyl methacrylate (MMA)
2	Initiator	Benzoyl peroxide (BPO)	2	Accelerator	N,N-Dimethyl para-toluidine (DMPT)/dimethyl para-toluidine (DMpt)
3	Radio-opacifier	Barium sulphate (BaSO_4_)/Zirconia (ZrO_2_)	3	Stabiliser	Hydroquinone
4	Antibiotics	Gentamycin			

## Data Availability

All the data used are in the manuscript.
